# Parental Awareness of Sexual Experience in Adolescent Boys With Autism Spectrum Disorder

**DOI:** 10.1007/s10803-015-2622-3

**Published:** 2015-10-19

**Authors:** J. Dewinter, R. Vermeiren, I. Vanwesenbeeck, Ch. Van Nieuwenhuizen

**Affiliations:** Tranzo, Scientific Centre for Care and Welfare, Tilburg University, PO Box 90153 (T618), 5000 LE Tilburg, The Netherlands; GGzE Centre for Child and Adolescent Psychiatry, PO Box 909 (DP1104), 5600 AX Eindhoven, The Netherlands; Department of Child and Adolescent Psychiatry, Curium-LUMC, PO Box 15, 2300 AA Oegstgeest, Leiden, The Netherlands; VU Medical Centre Amsterdam, PO Box 303, 1115 ZG Duivendrecht, The Netherlands; Interdisciplinary Social Science, Utrecht University, PO Box 80140, 3508 TC Utrecht, The Netherlands; Rutgers WPF, PO Box 9022, 3506 GA Utrecht, The Netherlands

**Keywords:** Autism spectrum disorder, Adolescence, Sexuality, Parental awareness

## Abstract

Parent report and adolescent self-report data on lifetime sexual experience in adolescents with ASD were compared in 43 parent-adolescent dyads. Parents tended to underestimate the lifetime sexual experience of their sons, particularly solo sexual experiences such as masturbation and experience with orgasm. Parental underestimation and unawareness of adolescents’ sexual experience may influence communication and education about sex and sexuality in families. These findings have implications for the interpretation of earlier research, based on parent and caregiver reports, on sexuality in adolescents with ASD.

## Introduction

A number of recent studies have asked adolescents and adults with autism spectrum disorders (ASDs) about their sexual experience and sexuality (Byers et al. 2012, 2013; Dewinter et al. 2015). These studies, which were based on self-report, showed that the lifetime sexual experience of high-functioning adolescent boys with ASD was comparable to that of their peers in the general population (Dewinter et al. 2015) or demonstrated healthy sexual functioning in adults with ASD (Byers et al. 2012, 2013). Sexual health (World Health Organization 2006) refers to a state of well-being and a positive, respectful, safe, and pleasurable approach of sexuality and sexual relationships. Earlier research based on parent (Holmes and Himle 2014) or caregiver reports (Hellemans et al. 2007), however, showed that parents and caregivers thought that adolescents with ASD had less experience of different sexual behaviours than was suggested by adolescents’ self-reports in a recent study (Dewinter et al. 2015). The difference between these two types of data may reflect parental underestimation of adolescents’ sexual experience. Research (e.g. Jaccard et al. 1998; Liddon et al. 2013; Mollborn and Everett 2010) demonstrated that parents of boys in the general population also underestimate the sexual experience (mainly sexual intercourse) of their adolescent children. So, parents of boys with ASD might not differ in their knowledge on the sexual experience of their sons compared to parents of non-ASD peers.

Since parents are the primary sex educators of their children (Sexuality Information and Education Council of the United States 2004), parents’ underestimation and unawareness of their sons’ sexual experience may have implications for communication and education related to these topics. Earlier research (Ballan 2012; Ruble and Dalrymple 1993) indicated that parents of children with ASD were less inclined to discuss issues of sexuality with their children if they believed that their child’s condition precluded a romantic relationship. Parents also reported uncertainty about what, when and how to tell their children about sex and sexuality (Nichols and Blakeley-Smith 2009). This uncertainty may be due to a combination of lack of knowledge about sexual development in adolescents with ASD, and lack of awareness or underestimation of their adolescent child’s sexual experience. Parental underestimation of, and unawareness about the sexual experience of adolescents with ASD may directly influence parent-adolescent communication about sex and sexuality, and the timing of parental sex education. Offering timely information about sexuality and how to deal with it can promote healthy sexual development in adolescents with ASD and help to prevent inappropriate or aversive sexual behaviours and experiences. Limited sexuality education might lead to unawareness in adolescents of conventions related to sexuality and relationships, to frustration (e.g. ineffective masturbation practices), and self-harm (e.g. excessive masturbation).

Sexuality development encompasses more than experience with solo and partnered sexual acts: it is about mind and body, individual experiences and attributes (e.g. desire, pleasure, identity, preference, fantasies, roles, and norms), relationships, and social influences (e.g. law, mores, and culture) (Tolman and Diamond 2014; WHO 2006). This study compared parent and adolescent reports of lifetime solo and partnered sexual behaviour in a sample of adolescent boys with ASD. We assumed that the boys would report more sexual experience than their parents reported being aware of.

## Method

### Participants

The inclusion criteria for adolescent participants were male sex; age 15–18 years; Dutch or Belgian cultural background and a diagnosis of an Autistic Disorder or Asperger’s Disorder. Adolescent participants also had to be attending mainstream school or score in the below average range or above on standardised intelligence measures (Full-Scale IQ > 70). Comorbid psychopathology, other than florid psychotic symptoms, was not an exclusion criterion. The parents of all adolescent participants could participate in the study. ASD features were assessed with the ADOS (Autism Diagnostic Observation Schedule), module 4 (fluent speech) (Lord et al. 1999). One hundred and forty-six boys and their parents received information on this study. The parents of 44 of the participating boys (*n* = 51) agreed to participate in this study and completed a questionnaire. One parent-adolescent dyad was excluded because the boy reported that he did not dare to answer the questions honestly, leaving a sample of 43 dyads (see Table 1 for participant characteristics). One or both parents completed the parental questionnaire. No additional information on the background of the parents is available.

**Table 1:** Sample characteristics (N = 43)

	M (SD) (range)	n	%
Age	16.67 (.81) (range: 15–18 years)		
Primary diagnosis (DSM IV-TR)			
Autistic disorder		18	25
Asperger’s syndrome		25	58
ADOS module 4 (n = 41)	8.29 (2.9) (range: 3–14)		
Above cut-off autism		16	37.2
Above cut-off autism spectrum		14	32.6
Below cut-off autism spectrum		11	25.6
Comorbidity			
Attention deficit disorders		12	28
Anxiety disorders		3	7
Learning disorders		3	7
Full Scale IQ (n = 39)	104.26 (15.87) (range: 76–142)		
Educational level			
Low (secondary education, prevocational level and lower)		26	60
High secondary education		17	40

Participants lived in the Netherlands or Belgium. In general the population of both countries has a liberal attitude to adolescent sexuality (de Looze et al. 2014), meaning that parents think about adolescent sexuality as a normative activity in romantic relationships. Comprehensive sex education is part of most school curricula.

### Materials

#### Self-Report Data

All boys answered nine questions on lifetime experience of romantic relationships and common solo and partnered sexual behaviours (Fortenberry 2013; Moore and Rosenthal 2006). These questions were part of an online questionnaire developed for the ‘Sex under the age of 25 II’ study (de Graaf et al. 2012). This questionnaire consisted of 177 questions relating to various aspects of sexual health. All nine questions followed a closed, multiple-choice answer format. The questions were formulated in simple language (‘Have you ever…’) and the relevant sexual behaviours were described in discrete boxes placed next to the questions. The response format for all questions was dichotomous (yes/no), except for the question on experience of orgasm (third alternative: I don’t know).

#### Parent Reports

Parents answered nine questions about their sons’ lifetime experience of various sexual behaviours, which were developed specifically for this study. The self-report questions on common sexual behaviours were adapted to be completed by parents (‘did your son ever…’ instead of ‘did you ever…’). All questions followed a closed, three-choice (yes; no; don’t know) format.

### Procedure

Following ethical review (Medical Ethical Committeeu reference NL34563.097.11 in the Netherlands; approval 4112 by the Institutional Review Board ZNA/OCMW in Antwerp, Belgium) participants were recruited from several institutions and schools between January 2012 and May 2013. Eligible participants and their parents received an information letter and leaflets on the study from the organisations and professionals working with them. None of the participants was dependent on the researchers for treatment. Parents and adolescents were invited to contact the researcher to indicate whether they were willing to participate. If no response was received within 10 days they were contacted again. A call for participants was also posted on the website of the Dutch Autism Association. All participating boys and parents gave written informed consent.

The participating boys could complete the online questionnaire in private and anonymously in whatever location they found convenient (at school; at home or another residence; at our centre); most chose to complete the questionnaire with a researcher nearby to answer questions if necessary. The researcher could not see the boys’ answers during completion. Some boys asked for help or preferred to have the questions read aloud by the researcher. Two boys preferred to complete the survey on their own at home. Participants received a €5 voucher after completing the survey. If possible, the ADOS was conducted in the same session, before the boy completed the questionnaire.

Parents could complete the parental questionnaire online at a secured webpage or in a paper-and-pencil version.

### Statistical Analysis

We report the number of adolescents who confirmed the different sexual experiences, agreement between parents and their sons separately on the occurrence and non-occurrence of the lifetime sexual experience, the proportion of parents who were ignorant of whether their son had or had not experienced each form of sexual behaviour, and parental awareness (sum of agreement on occurrence and non-occurrence). SPSS21 was used for data analysis.

The collected data were not suitable for further statistical analyses. The reported percentages do offer insight into differences between parent- and self-report.

## Results

As can be seen from Table 2, half of the parents reported that they did not know whether their sons had experienced masturbation or orgasm.

**Table 2:** Agreement on sexual experience between boys with ASD and their parents (N = 43 dyads)

Relational or sexual behaviour	Adolescent report		Parental report						Parental awareness^a^	
			Agreement on occurrence		Agreement on non-occurrence		Do not know			
	n	%	n	%	n	%	n	%	n	%
Masturbation	41	95.3	22	53.6	1	50	19	44.2	23	53.5
Orgasm	38	88.4	19	50	1	100	22	51.2	20	46.5
Relationship	32	74.4	28	87.5	10	91	1	2.3	38	88.4
French kissing	26	60.5	20	76.9	12	71	7	16.3	32	74.4
Petting above clothes	24	55.8	16	66.6	12	63	11	25.6	28	65.1
Penile/vaginal intercourse	12	27.9	8	66.7	22	71	12	27.9	30	69.8
Making love to a boy	1	2.3	0	0	39	93	3	7	39	90.7
Forcing someone else to do sexual things	2	4.7	1	50	35	85	7	16.3	36	83.7
Being forced to do sexual things	3	7	1	33.3	37	92	1	2.3	38	88.4

^a^Parents correctly aware of the presence or absence of sexual behaviour

Almost all boys reported that they had masturbated and the majority had experienced orgasm. About half the parents of adolescents who had solo sexual experience were aware of this. The majority of parents correctly reported that their son had had a relationship and or had experienced French kissing. About a third of the boys reported that they had had sexual intercourse with a girl. A quarter of the parents stated that they did not know if their son had experienced sexual intercourse. Eight out of twelve parents reported correctly that their son had had intercourse. The majority of parents reported same sex experience or lack thereof correctly. Overestimation by parents (i.e. false positives) was rare: two parents assumed incorrectly that their sons had experience with kissing, and one parent thought this about hugging. One in six parents stated that they did not know whether or not their son had forced someone to do sexual things, and most parents thought that their son had not suffered sexual victimisation. Only a small percentage of boys reported same-sex sexual experiences, sexual victimisation, or sexual coercion. Not all parents knew about of their sons’ negative sexual experiences (coercion and victimisation).

## Discussion

This study investigated agreement between parental and self-report on the lifetime sexual experience with common solo and partnered sexual acts in adolescent boys with ASD. The results confirm that boys with ASD report more sexual experience than their parents are aware of. Overall, a substantial proportion of parents was uncertain about the extent of their son’s sexual experiences or underestimated their son’s sexual experience, particularly with respect to solo sexual behaviours. There is no evidence that parents of adolescents with ASD differ from those of boys in the general population pertaining to their awareness of the sexual experience of their children. However, our insight in sexual functioning of adolescents with ASD is mostly based on parental, teacher, and caregiver reports (e.g. Hellemans et al. 2007, 2010; Mehzabin and Stokes 2011; Stokes and Kaur 2005) and might thus be biased. The results of this study have not only implications for the interpretation of earlier research but also for sex education.

Parents were less likely to report that they knew about their sons’ solo sexual experiences (masturbation; experiencing an orgasm) than about their partnered sexual behaviours (having been in a relationship; penile-vaginal intercourse). Parents often declared that they were uncertain about whether their sons masturbated, rather than incorrectly assuming that they did not. Parents’ uncertainty about their sons’ masturbation habits might indicate that these adolescents masturbated in private, as is socially appropriate. Masturbation plays an important role in the sexual development of boys (Robbins et al. 2011) and is one of the most common and earliest sexual experiences in male adolescent sexual development; low levels of parental knowledge about masturbation habits might reflect limited parent-adolescent discussion about masturbation. For many people, there is a taboo on talking about masturbation because of its undeniably sexual connotation. Frankel (2002) argued that parents were less inclined to discuss semenarche (first ejaculation) with their sons than menarche with their daughters due to the sexual connotations of semenarche. Although semenarche should not be confounded with masturbation it is possible that similar taboos influence parental discussion of both topics. Partnered sexual behaviours are possibly easier to discuss, because they can be related to safe sex and prevention of teenage pregnancy, i.e. discussed as a health issue rather than as a sex issue. Masturbation is more directly related to sexual pleasure. It might be valuable to offer adolescents boys with ASD timely and appropriate information about sexual arousal, sexual pleasure, and masturbation to support their sexual development. Holmes and Himle (2014) found that many parents did not discuss general aspects of sexuality and relationships, apart from issues related to abuse prevention, hygiene and privacy. Unfortunately our data do not reveal whether or not the parents in this study discussed these topics with their sons. The role of parent-adolescent communication about sex in the sexual development of adolescents with ASD is a subject for further research. Also, the data do not offer insight in the way the boys deal with masturbation. We do not know if there is a relationship between inappropriate masturbation practices and the awareness of parents.

Parent-adolescent agreement about sexual experiences was higher with respect to romantic relationships and partnered sexual experiences than solo sexual experiences. The majority of the boys with ASD in this study had been in a romantic relationship, and most parents were aware of this; nevertheless about a third of parents underestimated their sons’ partnered sexual experience. The underestimation of sexual experience of their sons’ might reflect limited discussion of sexual experiences between adolescents and parents. It is also possible that assumptions about their sons’ lack of sexual experience temper parents’ inclination to discuss partnered sexual behaviours with their sons. The taboo on discussing romantic relationships is possibly less strong than the taboo on talking about the sexual aspects of relationships. The higher level of agreement about partnered sexual experience might also be explained by the generally lower frequencies of partnered experiences. Given parental underestimation, the probability of agreement on the absence of a behaviour is higher in the case of low-frequency behaviours (Mollborn and Everett 2010). It would be interesting to examine parent-adolescent agreement on the partnered sexual experiences of older boys with ASD as it is possible that a higher proportion of them will have had partnered sexual experiences.

The number of boys who reported having forced someone else to do sexual things or having been forced to do sexual things themselves was low. Slightly more parents stated that they did not know if their son had coerced someone into sexual behaviour than reported that they did not know if their son had been victimised sexually. This finding should be interpreted with care, given the exploratory nature of this study; however parents could have doubted about the possibility that their sons coerced others to sexual behaviours.

Earlier research (Ballan 2012; Nichols and Blakeley-Smith 2009) suggested that parents do not know what to expect when it comes to the sexual development of adolescent children with ASD and hesitate to discuss issues related to sex and sexuality when they are unsure whether the adolescent has any sexual interest. It is possible that adolescents react negatively or hesitantly when their parents initiate discussions about sex and sexuality and this might further enhance parents’ uncertainty about how to handle the issue. Given that only limited evidence is available on the sexual development of children and adolescents with ASD, the underestimation of sexual experience seen in this study might be related to a lack of sex communication and education among a significant proportion of adolescents with ASD. Research evidence and clinical opinion agree that sex education delivered by parents is important (Ballan 2012; Hellemans et al. 2007; Mehzabin and Stokes 2011; Nichols and Blakeley-Smith 2009; Stokes and Kaur 2005), so it is advisable to help parents fulfil this duty by providing information appropriate to the age and physical maturity of their child. There is still no systematic evidence about the prevalence of abnormal sexual development in adolescents with ASD; it is therefore possible that features of ASD might increase the probability of a child finding the physical changes and development of sexual interest associated with sexual maturation a negative experience. Similarly, features of ASD may increase the risk of developing inappropriate sexual behaviours. In daily clinical practice we meet adolescents who develop specific, sometimes inadequate, masturbation techniques or paraphilic arousal patterns that cause concern when they come to light. There is also no evidence on the prevalence of paraphilias or problems with masturbation in boys with ASD; however early and sensitive attention to sexual arousal and masturbation may promote healthy sexual development. It might be a challenge for parents to understand and discuss sexuality development in adolescent with ASD, and to deal with seemingly inappropriate sexual behaviours. Hence, professional support and information should be available for parents.

Finally, parents’ underestimation and ignorance of the extent of the lifetime sexual experience of adolescent sons with ASD has implications for the interpretation of research based on parent or caregiver reports. Future research on sexuality in people with ASD should include self-report data in order to get more insight into development in this domain. Additional research based on self-report data could improve our understanding of sexual development in adolescents with ASD.

### Strengths, Limitations, and Further Research

This is the first comparison of parental reports and self-reports of the lifetime sexual experience of boys with ASD in a sample of parent–child dyads and provides additional insight into earlier studies based on parent and caregiver reports of sexual behaviours in adolescents with ASD. Nevertheless the limitations of the study should be borne in mind. First, possible selection bias and the participant profile (the sample was limited to adolescents with high-functioning autism) limit the generalizability of our findings. Second, it has been reported that mothers and fathers communicate differently about sex and sexuality with their children (Diiorio et al. 2003) and we do not have data on which parent or parents completed the questions about adolescent sexual experience in our study. Third, this study only focused on lifetime experience with the most common solo and partnered sexual acts, leaving questions on different other aspects relating to sexuality still unanswered.

Our results and the limitations of the study raise issues for further research. First, this study should be replicated in other samples of adolescent boys and girls with ASD to confirm and refine our findings. In addition, further research should look for behavioural cues to detect adolescents’ problems or concerns about sex and sexuality, which should trigger additional support or early intervention. Finally, little is known about the variables, for instance parent–child communication about sex and sexuality, which influence parental awareness of the sexual behaviour of adolescents with ASD.

## Conclusion

Parents play a critical role in education about sex and sexuality, although they are often uncertain about how, when and what information to provide or discuss (Ballan 2012; Holmes and Himle 2014; Nichols and Blakeley-Smith 2009). Comprehensive sex education, including a variety of issues relating to sexuality including solo and partnered sex (SIECUS 2004) is likely to play an important role in promoting healthy sexual development and preventing harmful or aversive sexual experiences in adolescents with ASD, as it does in other adolescents. Several authors (e.g. Attwood et al. 2014; Dekker et al. 2015; Hénault 2005) made suggestions on sexuality education and developed programs attuned to adolescents with ASD. This study showed that parents tend to be uncertain about, or underestimate the extent of their adolescent’s sexual experience; this suggests they would benefit from receiving information about sexual development in adolescents with ASD. Such information should be made more available and parents should also have access to support to help them make decisions about when and how to discuss issues of sex and sexuality .

## References

[CR1] Attwood T, Hénault I, Dubin N (2014). The autism spectrum, sexuality and the law: What every parent and professional needs to know.

[CR2] Ballan MS (2012). Parental perspectives of communication about sexuality in families of children with autism spectrum disorders. Journal of Autism and Developmental Disorders.

[CR3] Byers ES, Nichols S, Voyer SD (2013). Challenging stereotypes: Sexual functioning of single adults with high functioning autism spectrum disorder. Journal of Autism and Developmental Disorders.

[CR4] Byers ES, Nichols S, Voyer SD, Reilly G (2012). Sexual well-being of a community sample of high-functioning adults on the autism spectrum who have been in a romantic relationship. Autism.

[CR5] De Graaf H, Kruijer H, van Acker J, Meijer S (2012). Seks onder je 25e: Seksuele gezondheid van jongeren in Nederland anno 2012 [Sex under the age of 25: Sexual health among youth in The Netherlands in 2012].

[CR6] De Looze M, Constantine N, Jerman P, Vermeulen-Smit E, Ter Bogt T (2014). Parent-adolescent sexual communication and its association with adolescent sexual behaviors: A nationally representative analysis in the Netherlands. Journal of Sex Research.

[CR7] Dekker LP, Van Der Vegt EJM, Visser K (2015). Improving psychosexual knowledge in adolescents with autism spectrum disorder: Pilot of the tackling teenage training program. Journal of Autism and Developmental Disorders.

[CR8] Dewinter J, Vermeiren R, Vanwesenbeeck I, Lobbestael J, Van Nieuwenhuizen Ch (2015). Sexuality in adolescent boys with autism spectrum disorder: Self-reported behaviours and attitudes. Journal of Autism and Developmental Disorders.

[CR9] Diiorio C, Pluhar E, Belcher L (2003). Parent–child communication about sexuality. Journal of HIV/AIDS Prevention & Education for Adolescents & Children.

[CR10] Fortenberry JD, Bromberg DS, O’Donohue WT (2013). Sexual development in adolescents. Handbook of child and adolescent sexuality: Developmental and forensic psychology.

[CR11] Frankel L (2002). “I’ve never thought about it”: Contradictions and taboos surrounding American males’ experiences of first ejaculation (semenarche). The Journal of Men’s Studies.

[CR12] Hellemans H, Colson K, Verbraeken C, Vermeiren R, Deboutte D (2007). Sexual behavior in high-functioning male adolescents and young adults with autism spectrum disorder. Journal of Autism and Developmental Disorders.

[CR13] Hellemans H, Roeyers H, Leplae W, Dewaele T, Deboutte D (2010). Sexual behavior in male adolescents and young adults with autism spectrum disorder and borderline/mild mental retardation. Sexuality and Disability.

[CR14] Hénault I (2005). Asperger’s syndrome and sexuality: From adolescence through adulthood.

[CR15] Holmes LG, Himle MB (2014). Brief report: Parent–child sexuality communication and autism spectrum disorders. Journal of Autism and Developmental Disorders.

[CR16] Jaccard J, Dittus PJ, Gordon VV (1998). Parent-adolescent congruency in reports of adolescent sexual behavior and in communications about sexual behavior. Child Development.

[CR17] Liddon N, Michael SL, Dittus P, Markowitz LE (2013). Maternal underestimation of child’s sexual experience: Suggested implications for HPV vaccine uptake at recommended ages. Journal of Adolescent Health.

[CR18] Lord C, Rutter M, Dilavore PC, Risi S (1999). Autism diagnostic observation schedule: Manual.

[CR19] Mehzabin P, Stokes M (2011). Self-assessed sexuality in young adults with high-functioning autism. Research in Autism Spectrum Disorders.

[CR20] Mollborn S, Everett B (2010). Correlates and consequences of parent-teen incongruence in reports of teens’ sexual experience. Journal of Sex Research.

[CR21] Moore S, Rosenthal D (2006). Sexuality in adolescence: Current trends.

[CR22] Nichols S, Blakeley-Smith A (2009). “I’m not sure we’re ready for this …”: Working with families toward facilitating healthy sexuality for individuals with autism spectrum disorders. Social Work in Mental Health.

[CR23] Robbins CL, Schick V, Reece M, Herbenick D, Sanders S, Dodge B, Fortenberry JD (2011). Prevalence, frequency, and associations of masturbation with partnered sexual behaviors among US adolescents. Archives of Pediatrics and Adolescent Medicine.

[CR24] Ruble LA, Dalrymple MS (1993). Social/sexual awareness of persons with autism: A parental perspective. Archives of Sexual Behavior.

[CR25] SIECUS. (2004). *Guidelines for comprehensive sexuality education: Kindergarten through 12th grade*, 3rd ed. Retrieved from http://www.siecus.org/_data/global/images/guidelines.pdf

[CR26] Stokes M, Kaur A (2005). High-functioning autism and sexuality: A parental perspective. Autism.

[CR27] Tolman DL, Diamond LM, Tolman DL, Diamond LM (2014). Introduction. APA handbook of sexuality and psychology (pp. xix–xxviii).

[CR28] WHO (2006). Defining sexual health: Report of a technical consultation on sexual health, 28–31 January 2002, Geneva.

